# Environmental Contaminants Modulate Breast Cancer Development and Outcome in *TP53* p.R337H Carriers and Noncarriers

**DOI:** 10.3390/cancers14123014

**Published:** 2022-06-19

**Authors:** Viviane K. Q. Gerber, Mariana M. Paraizo, Humberto C. Ibañez, José C. Casali-da-Rocha, Emilia M. Pinto, Diancarlos P. Andrade, Marilea V. C. Ibañez, Heloisa Komechen, Mirna M. O. Figueiredo, Gislaine Custódio, Carmem M. C. M. Fiori, José H. G. Balbinotti, Jeanine M. Nardin, Thais A. Almeida, Olair O. Beltrame, Paula A. Yamada, Gabriel S. de Fraga, Lucas L. de Brito, Jamylle Martins, Viviane S. Melanda, Otavio A. B. Licht, Victoria Z. Teixeira, Sandy K. S. Pinho, Silvia Bottini, Enzo Lalli, Gerard P. Zambetti, Bonald C. Figueiredo

**Affiliations:** 1 Pelé Pequeno Príncipe Research Institute, Avenida Silva Jardim, 1632, Água Verde, Curitiba 80250-200, PR, Brazil; vivianekg@yahoo.com.br (V.K.Q.G.); mary_paraizo@hotmail.com (M.M.P.); humberto.ibanez@gmail.com (H.C.I.); diancarlospa@gmail.com (D.P.A.); marilea.ibanez@pelepequenoprincipe.org.br (M.V.C.I.); heloisakomechen@gmail.com (H.K.); jose.balbinotti98@gmail.com (J.H.G.B.); 2 Faculdades Pequeno Príncipe, Avenida Iguaçu, 333, Rebouças, Curitiba 80230-020, PR, Brazil; 3 Universidade Estadual do Centro-Oeste, UNICENTRO, Alameda Élio Antonio Dalla Vecchia, 838, Vila Carli, Guarapuava 85040-167, PR, Brazil; vzteixeira@yahoo.com (V.Z.T.); sandyksoares@hotmail.com (S.K.S.P.); 4 Erasto Gaertner Hospital, Rua Dr. Ovande do Amaral, 201, Jardim das Américas, Curitiba 81520-060, PR, Brazil; casali.rocha@accamargo.org.br (J.C.C.-R.); jnardin@erastogaertner.com.br (J.M.N.); thais@iop.com.br (T.A.A.); ocbeltrame@gmail.com (O.O.B.); paulayamada@hotmail.com (P.A.Y.); gabrieldefraga96@gmail.com (G.S.F.); lucasluann0@gmail.com (L.L.B.); martinsjamylle@gmail.com (J.M.); 5 Faculdade de Medicina, Pontifícia Universidade Católica do Paraná (PUCPR), Rua Imaculda Conceição, 1155, Prado Velho, Curitiba 80215-901, PR, Brazil; 6 Faculdade de Medicina da Universidade Positivo, Rua Professor Pedro Viriato Parigot de Souza, 5300, Ecoville, Curitiba 81280-330, PR, Brazil; 7 Department of Pathology, St. Jude Children’s Research Hospital, 262 Danny Thomas Pl, Memphis, TN 38105, USA; emilia.pinto@stjude.org (E.M.P.); gerard.zambetti@stjude.org (G.P.Z.); 8 Centro de Genética Molecular e Pesquisa do Câncer em Crianças-CEGEMPAC, Universidade Federal do Paraná, Avenida Agostinho Leão Junior, 400, Alto da Glória, Curitiba 80030-110, PR, Brazil; mirnafigueiredo@hotmail.com (M.M.O.F.); custodio.gislaine@gmail.com (G.C.); 9 União Oeste Paranaense de Estudos e Combate ao Câncer–UOPECCAN, Rua Itaquatiaras 769, Santo Onofre, Cascavel 85.806-300, PR, Brazil; carmem.fiori@uopeccan.org.br; 10 Departamento de Vigilância Epidemiológica, Secretaria do Estado da Saúde do Paraná, Rua Piquiri, 17, Rebouças, Curitiba 80230-140, PR, Brazil; vivianes@sesa.pr.gov.br; 11 Instituto de Terras, Cartografia e Geologia, Rua Desembargador Motta, 338, Mercês, Curitiba 80430-232, PR, Brazil; otavio.licht@gmail.com; 12 Center of Modeling, Simulation & Interaction, Université Côte d’Azur, 1361 Route des Lucioles, 06560 Valbonne, France; silvia.bottini@univ-cotedazur.fr; 13 Institut de Pharmacologie Moléculaire et Cellulaire CNRS, Valbonne, 660 Route des Lucioles, 06560 Valbonne, France; lalli@ipmc.cnrs.fr

**Keywords:** breast cancer, *TP53* p.R337H, *XAF1* p.E134*, environment, pesticides

## Abstract

**Simple Summary:**

Although an inherited *TP53* p.R337H mutation alone plays a critical role in breast cancer (BC) development, exposure to pesticides, industry contaminants, and air pollutants, along with poor lifestyle choices, are associated with the development of BC. The aim of our study was to assess the joint effect of the reduced tumor suppression activity of *TP53* p.R337H and the environmental factors that may modulate individual susceptibility to BC. We evaluated the environmental differences and proportional distribution of *TP53* p.R337H carrier and noncarrier women in three subregions of Southern Brazil to estimate the prevalence, risk, and/or mortality rate of BC. We found that a p.R337H mutation is more likely to cause BC in individuals living in a heavily polluted environment. The impact of environmental contaminants can be reduced by screening, genetic testing, medical improvements in early BC detection, and promoting healthier lifestyle practices.

**Abstract:**

Two major concerns associated with cancer development in Paraná state, South Brazil, are environmental pollution and the germline *TP53* p.R337H variant found in 0.27–0.30% of the population. We assessed breast cancer (BC) risk in rural (C1 and C2) and industrialized (C3) subregions, previously classified by geochemistry, agricultural productivity, and population density. C2 presents lower organochloride levels in rivers and lower agricultural outputs than C1, and lower levels of chlorine anions in rivers and lower industrial activities than C3. *TP53* p.R337H status was assessed in 4658 women aged >30 years from C1, C2, and C3, subsequent to a genetic screening (Group 1, longitudinal study). BC risk in this group was 4.58 times higher among *TP53* p.R337H carriers. BC prevalence and risk were significantly lower in C2 compared to that in C3. Mortality rate and risk associated with BC in women aged >30 years (*n* = 8181 deceased women; Group 2) were also lower in C2 than those in C3 and C1. These results suggest that environmental factors modulate BC risk and outcome in carriers and noncarriers.

## 1. Introduction

Various cancer profiles have been shown to be associated with environmental hazards [1]; however, those associated with mutated tumor-suppressor genes have not been reported. Furthermore, the interactions among a wide range of environmental stressors and genetic variants and their effects on tumor penetrance are not well understood. 

The germline *TP53* p.R337H variant (rs121912664) is rare globally, yet common in South [2,3,4,5] and Southeast Brazil [6]. This p.R337H founder variant [7] co-segregates with the nonsense p.E134* variant in the putative tumor suppressor XIAP-associated factor 1 gene (*XAF1* p.E134*) (69% p.R337H alleles), which may contribute to a variable tumor phenotype [8]. 

The reported frequencies of p.R337H among individuals with breast cancer (BC) from South and Southeast Brazil range from 0.5% to 8.6% [9,10,11,12,13]. The basis for this wide range of frequencies may be attributed to study design (e.g., patient selection criteria), along with a combination of genetic, epigenetic, or environmental factors. 

Assessment of the penetrance of the *TP53* p.R337H haplotype is challenged by significant variations in environmental exposure and other unidentified genetic and/or epigenetic variants in South and Southeast Brazilian populations. This may explain why more than 50% of families carrying the p.R337H variant in the Paraná state [3,4,5] do not fit the more flexible clinical criteria for classical Li–Fraumeni syndrome (LFS) [14]. 

One major challenge preventing efficient sustainable development in industrial and agriculture sectors is a lack of necessary environmental management for tackling environmental degradation. Evidence of BC risk in Paraná and other Brazilian states has been reported for pesticides used in agriculture [15]. Additionally, in a rural region of Australia with high levels of organochlorines (OCs), higher rates of female BC have been reported compared with rates in the metropolitan area of Melbourne [16]. In another cohort, spouses of private applicators provided information about their own use of pesticides, including seven OCs, and they found an increased risk of estrogen-receptor-negative/progesterone-receptor-negative BC associated with dieldrin exposure in North Carolina and Iowa [17]. A multi-case control study carried out in Spain found an increased risk of BC in women living near specific industrial installations [1]. In addition, air pollution—mostly caused by fumes from cars and smoke from burning fuels, such as wood or coal, industrial or agricultural emissions, and residential heating and cooking—has significantly risen, particularly in countries with large populations. Cohort-specific associations based on pooled cohort-specific estimates reported an association between long-term exposure to air pollution and the incidence of postmenopausal BC in a large, multicenter European study [18]. Thus, environmental stressors potentially implicated in BC development are found in different levels and types in both rural and metropolitan areas.

In this study, we focused on the Paraná state, which has previously been divided into subregions with small and large municipalities [19]. The chloride level was measured in catchment basins, where the concentrations were lower in the central-southern (C2) subregion compared to the northern-western subregion with high-yield crops (C1) and the eastern-southern subregion strongly industrialized with a high population density (C3). The sampling period for the chemical analyses in the catchment basins (2006–2010), agriculture output, and congenital malformations analyses in C1, C2, and C3 [19] coincides with most examined BC cases. 

The primary objectives of the current study were evaluated in two groups of BC patients. Group 1 included living and deceased women identified through a neonatal screening program, and Group 2 included only deceased women, all aged 30 years and older. Prospective and retrospective BC cases in women detected in 373 families carrying the p.R337H variant from a neonatal screening (Group 1) were studied according to the environmental characteristics of the three subregions. The findings were validated by comparing with the mean BC mortality rates by subregion (cross-sectional study, Group 2, Paraná state Public Database comprising all 399 municipalities). The prevalence of BC in our neonatal screening cohort of *TP53* p.R337H carriers and noncarriers (2006–2018, Group 1) and the BC mortality rates reported for other unknown women from the public database (Group 2) were evaluated in each subregion to estimate BC risk. As secondary objectives, we also examined whether *TP53* p.R337H carriers in Group 1 present the *XAF1* p.E134* variant, as previously reported in children without cancer [8]. Additionally, BC patients, unselected for family history of cancer (i.e., unknown cancer risk), admitted to a cancer reference hospital (Erasto Gaertner Hospital, Group 3) were tested for *TP53* p.R337H and *XAF1* p.E134* to obtain the genotype and allele frequencies of theses mutations in BC in Paraná state. Given the likely association of BC risk with obesity, we also evaluated the body mass index (BMI) in Group 1.

## 2. Materials and Methods

### 2.1. Rationale for Classification of the Paraná State into Three Subregions

We previously classified Paraná state into three subregions (C1, C2, and C3) according to the chloride levels in rivers (presumably originated from chlorine-containing products), levels of organochlorines, agriculture production per km^2^, and/or industrial activities combined with high population density [19]. Chloride concentrations was examined in 63% (465/736) of the catchment basins of Paraná state (199.727 km^2^ surface). River water was collected from a specific point in the basin where the tributary flows into the main river. The water sample was vacuum-filtered, bottled, and stored at −20 °C. Chloride was quantified by ion chromatography. The chloride concentrations determined in the basins that intersect the municipality area were weighted to estimate the average of these concentrations in each municipality. Municipalities were grouped according to chloride level (below or above 1.81 mg/L) and according to the predominant regional activity (agricultural or industrial, including oil refineries, extractive, manufacturing, and metallurgical industries). 

### 2.2. Long-Term Follow-Up of Women Negative and Positive for the Germline TP53 p.R337H and XAF1 p.E134* Variants in the Three Subregions of Paraná State 

Over 600 families from a longitudinal study were recruited for follow-up to date through two neonatal screenings; however, only 373 families identified during neonatal screening 1 [3] met the criteria for this study (more than 5 years follow-up, complete cancer history, pedigree with four or more generations, over 80% of genotyped individuals). Our multidisciplinary approach involved environmental investigators, oncologists, genetic counselors, psychologists, molecular biologists, and geoprocessing specialists. The selected cohort (2006–2018) included 4165 p.R337H carriers of all ages, males and females, including those who were identified in extended families. p.R337H noncarrier women aged 30 years or more (*n* = 3562) and p.R337H carrier women (*n* = 1096) were considered eligible for the determination of BC prevalence. These women were living in the subregions (C1, C2, or C3) for at least the last 10 years.

The follow-up protocol offered for these families carrying the *TP53* p.R337H variant was based on four objectives: (1) provision of educational and genetic counseling; (2) acquisition of pedigrees, family cancer history, demographic information, and provision of DNA tests for all family members; (3) surveillance for early diagnosis and treatment of adrenocortical carcinoma among children; (4) provision of other pediatric or adult patient referral to specialized cancer centers. 

Prevalence rates for BC were calculated, separating individuals who were negative and positive for the *TP53* p.R337H variant and excluding families with other familial cancer predisposition syndromes (confounders such as BRCA1/BRCA2 variants or families presenting almost exclusively with BC and/or ovarian cancer). The families in this cohort had been living in one of the 175 municipalities distributed among the three subregions (C1, C2, or C3), excluding the carrier women living in other regions. After genetic counseling, participants were followed biannually from 2006 to the end of 2018 to provide support for rapid cancer diagnosis and treatment. Individuals who refused to participate, did not have demographic or clinical data, failed to follow up, and those with germline BRCA1/BRCA2 or presenting in families with three or more cases of BC and/or ovary cancer (without other tumors), without documented clinical or pathological diagnosis, and/or belonging to subregion C0 (not previously tested for chloride) were excluded (*n* = 25). The participants were recruited after signing a consent form, approved by the Hospital de Clínicas of Federal University of Paraná and Hospital Pequeno Príncipe Ethics Committee in 2005 (CAA: 0023.0.208.000-05) and the Hospital Pequeno Príncipe Ethics Committee in 2009 (CAAE 0612.0.015.000-08). 

### 2.3. Body Mass Index (BMI) in Breast Cancer Women 

BMI was evaluated in p.R337H carrier and noncarrier women in the neonatal screening cohort (Group 1) living in subregions C1, C2, or C3. This study was not designed to find BMI differences between the subregions, nor between carriers and noncarriers, given the constant communication with all families to preserve lifestyle and reduce exposure to environmental pollution.

### 2.4. Cross-Sectional Analysis of Breast Cancer Average Age-Specific Mortality Rate (AASMR) in the Three Subregions of Paraná State (Group 2)

The BC AASMR calculated per 100,000 women over 30 years of age was estimated from the Federal Ministry of Health registry [20]. We computed the average BC cases by dividing the sum of cases in each municipality that make up the subregion by the 11-year period (2005–2015). This annual average number of BC cases was divided by the 2010 census population (central population between 2005 and 2015) of the respective subregion (C1, C2, or C3) to obtain the AASMR. The speed of population growth in this state has been constant in the last 40 years [20].

### 2.5. Frequency of Germline TP53 p.R337H and XAF1 p.E134* Variants in Women with BC from a Paraná State Public Cancer Hospital (Group 3) from All Subregions of Paraná

Haplotypes were determined using DNA samples of blood and tumors from women admitted with BC to the Erasto Gaertner Hospital (EGH) in Curitiba (2015–2018), randomly selected after signing a consent form approved by the local Ethics Committee (CAAE: 51367415.2.1001.0098). These patients presented tumors at various stages and degrees of malignancy without any knowledge about where and how long they lived in each subregion. Therefore, they were excluded from our environmental analysis. Admittance is usually controlled by the public system (SUS), and referral according to surgical or clinical complexity. 

### 2.6. TP53 p.R337H and XAF1 p.E134* Genotyping

*TP53* p.R337H genotyping was performed as previously reported [21]. The *XAF1* p.E134* variant was detected using TaqMan allelic discrimination assays (Applied Biosystems, Foster City, CA, USA), as previously reported [8]. The *TP53* p.R337H alone or expressed along with the *XAF1* p.E134* variant and their frequencies were assessed in Groups 1 and 3.

### 2.7. Geographic Visualization and Statistical Analysis

Since most younger generations have a lower number of adult women susceptible to BC than older generations, BC cases from the third to the last generation were combined as generation III, to increase statistical power in the age estimation per generation. Geographic visualizations were performed using QGIS 2.14 [22] and PostGIS 2.4 [23], and statistical analyses using R 3.4.1 [24]. Chi-squared tests were used to evaluate the relationships between two categorical variables [25,26]. The Kaplan–Meier nonparametric estimator [27] was used to estimate the age of BC occurrence, and log-rank tests [28] were used to assess significant differences in the probability curves for cancer onset. Estimated marginal means from a linear model and pairwise comparisons via Tukey tests [29] were used to evaluate differences in mean BMIs between carriers and noncarriers and among subregions. Results with *p* values < 0.05 were considered statistically significant.

## 3. Results

### 3.1. Environmental Factors

Significantly higher chloride concentrations (≥1.81 mg/L) were observed in the eastern and southern subregion (C3 subregion, including large population density and industrialized cities, and hence the lowest agriculture output) and in the northern and western subregion (C1 subregion, with the strongest agricultural output per square kilometer). The lowest chloride levels (<1.81 mg/L) were identified in the state center and extended southward (C2 subregion, characterized by agricultural outputs per square kilometer inferior to C1 and superior to C3). C1 was strongly associated with high-yield crops and presented high levels of three OCs, i.e., p,p′-DDT, p,p′-DDE, and p,p′-DDD; C2 was less influenced by agriculture (lower levels of OCs) and industrial activities [19].

### 3.2. Breast Cancer Chracteristics

Benign breast tumors were excluded, and the malignant tumors were classified according to their localization and histopathological characteristics [30]. Complete histological analysis was available for 363 tumors at tumor stage 1 (81, 22%), 2 (159, 44%), 3 (86, 24%), and 4 (33, 9%). These tumors were categorized by immunohistochemistry and molecular subtypes using CISH (local-regional recurrence gene signatures) as follows: 256 (71%) tumors categorized as luminal A and B; 256 (71%) as RE+/RP+; 66 (18%) as HER2+; and 67 (18%) triple negative. Analyses of each tumor subtype did not yield significant differences between haplotypes *TP53* p.R337H and/or *XAF1* p.E134* and noncarriers.

### 3.3. BC Risk among Women with Negative and Positive TP53 p.R337H Variant (Group 1)

The median follow-up time was 9.84 years (range, 9.67–10.01 years, from 2006 to 2018), while no other BC cases or carriers were included after this period to avoid large differences in timeline in relation to Groups 2 and 3. We limited the period to 2018 since it was closer to the sampling period for chemical analyses in the catchment basins (2006–2010) and the population census (2010). The 8-year difference corresponded to the approximate half-lives of OCs (p,p′-DDT, p,p′-DDE, and p,p′-DDT), while all other environmental conditions remained unchanged. Malignancy was ascertained by pathology or clinical reports. We identified 61 BC cases among 3562 women negative for p.R337H and 81 BC cases among 1096 p.R337H carrier women. Only 14 deceased BC cases in Group 1 overlapped with Group 2. The percentages of *TP53* p.R337H carrier and noncarrier women, as well as all BC cases in women older than 30 years, across all generations of the Paraná cohort are shown for all three subregions and according to generation (Table 1). BC was 4.58 times more frequent (confidence interval (CI): 3.2–6.5, *p* < 0.001) among p.R337H carriers (81/1096) than noncarriers (61/3562) in the cohort derived from the neonatal screening (Group 1).

**Figure 1:**
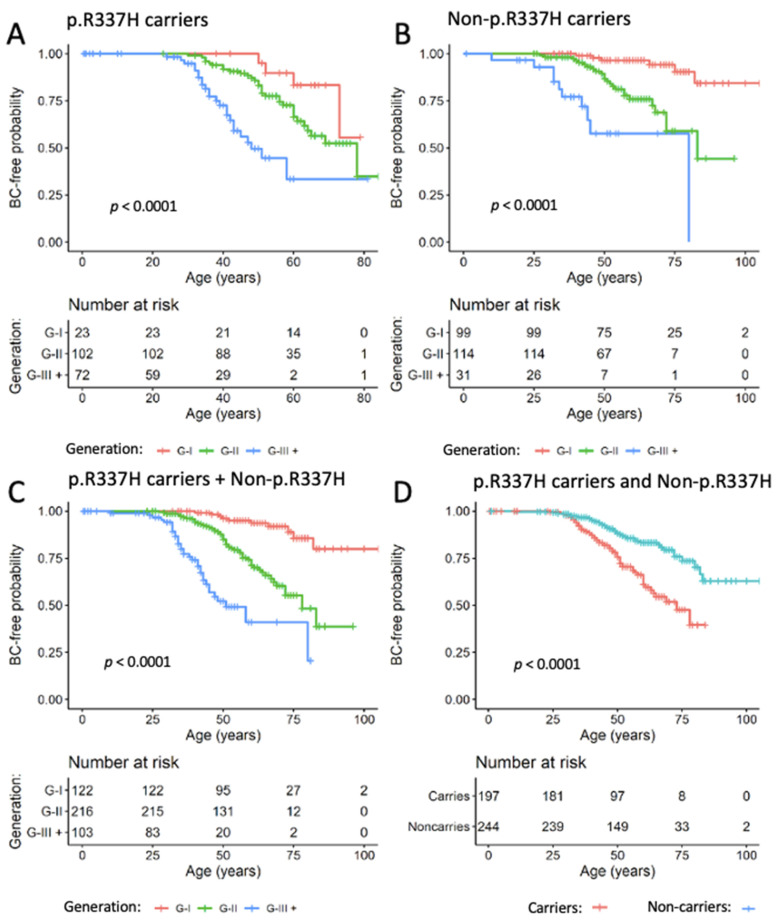
BC-free probabilities among *TP53* p.R337H carriers and noncarriers (Group 1). Censoring (vertical line) is indicated by vertical marks. BC occurrences in three generations (last two or three were grouped as the third generation) are shown in panels (**A**–**C**). Panel (**D**) (grouping all generations) shows younger age of BC among p.R337H carriers than noncarriers.

Two subgroups of participants are shown in Figure 1, carrier (*n* = 197) and non-carrier (*n* = 244) referred to private clinics or public hospitals, which included 59 carriers and 40 non-carriers with BC (age > 30 years). The women without malignant breast tumors were excluded (vertical lines shown in the curves) because they had benign breast tumor, other type of cancer or other diseases, or remained unaffected and were dismissed (some of them were reintegrated to the follow-up). The other BC cases (22 carriers and 21 non-carriers) were diagnosed before family enrollment to the cohort (i.e., before identification of the newborn proband). Over the last decades, there have been more BC cases diagnosed in younger women (*p* < 0.0001, Figure 1A–C), more significantly among p.R337H carriers (*p* < 0.0001, Figure 1D), with a median age over 40 years. An earlier age of cancer onset in the third generation (mean age: 41.2 years) was not exclusive to *TP53* p.R337H carriers; noncarriers with BC showed a similar age reduction in the last generation (mean age: 41.7 years). 

The risk ratio (RR) of BC in each exposed group in all three subregions was calculated for p.R337H carriers and noncarriers of the Group 1 cohort (Table 2). The risk of BC among p.R337H carrier women was 81% greater in C3 than C2 (*p* = 0.02). This difference was not associated with a higher number of BC cases expressing p.R337H and *XAF1* p.E134*, as this combination was equally proportional between C1, C2, and C3. Subdividing women aged ≥30 years of the Paraná newborn cohort (Group 1) per subregion (p.R337H carriers and noncarriers), C1 (3.2%, 31/945) and C3 (3.9%, 63/1599) were associated with a higher probability of BC development than C2 (2.2%, 48/2114; Table 1). Thus, considering all patients in the Paraná newborn cohort, positive and negative for p.R337H, BC risk in subregion C3 was 73% higher than that in C2 (*p* = 0.003).

The number of BC patients per subregion depends on the number of exposed carriers and noncarriers in each subregion (evaluated only for Group 1, i.e., the cohort from neonatal screening), and comparisons were made only between two age groups in the same subregion (same number of exposed carriers or noncarriers). We found no significant differences between age groups (<45 years vs. ≥45 years in the p.R337H carriers in each subregion (53% vs. 47% in C1; 44% vs. 56% in C2; 49% vs. 51% in C3). A slightly higher proportion of cases was found among noncarriers ≥45 years in C1 (58%), C2 (64%), and C3 (59%) (Table 3). For Group 3 (hospital database), we found 114 (22%, 114/506) and 392 (78%, 392/506) BC cases among p.R337H noncarriers in age groups <45 years and ≥45 years, respectively. Among the eight patients positive for the germline p.R337H in Group 3, three were diagnosed before 45 years of age (3/8, 38%), and five at or after 45 years of age (4/7, 62%) (Table 3). Individual differences in the number or proportion of cases between the two age groups at each subregion were not statistically significant. 

### 3.4. BC Age-Specific Mortality Rates in Each Subregion (Group 2)

**Figure 2:**
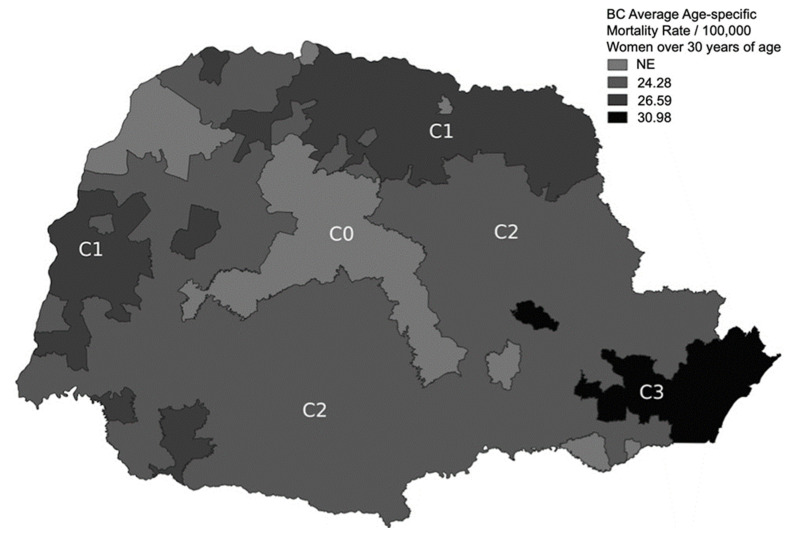
Breast cancer age-specific mortality rate (2005–2015) per 100,000 women over 30 years of age (Group 2). C0 was not evaluated.

According to the 2010 census, the total numbers of women 30 years or older were statistically balanced among C1 (*n* = 759.083), C2 (*n* = 1033.835), and C3 (*n* = 831.323). However, as shown in Table 4 and Figure 2, the numbers of municipalities were different, i.e., 114 in C1, 200 in C2, and 19 in C3 (including the capital, Curitiba). Thus, most towns in C1 and C2 are typically small rural cities. The C2 subregion, including mainly small rural cities, had the lowest mean BC mortality rate (2005–2015) of the three subregions: 24.28 per 100,000 inhabitants compared with 26.59 and 30.98 per 100,000 inhabitants in C1 and C3, respectively. Based on the calculated relative risk, C3 had a 27.6% higher risk for BC than C2 (*p* < 0.001, Table 4, Figure 2). The lowest Group 2 mortality rate (24.28/100.000) was found in C2, which was significantly lower than that in C3 (30.98/100.000, RR = 1.276 [CI, 1.21–1.34], *p* < 0.001) and lower than that in C1 (26.59/100.000, RR = 1.095 [CI, 1.036–1.158], *p* < 0.002).

### 3.5. TP53 p.R337H and XAF1 p.E134* Allele Percentages in Women with BC 

Group 1 BC cases (*n* = 61) showed no R337H noncarriers who were positive for *XAF1* p.E134*, whereas 77% of the p.R337H carriers (62/81) also presented p.E134* (13 in C1, 20 in C2, and 29 in C3, corresponding to 76%, 79%, and 75% of BC in each subregion, respectively). The difference in all p.R337H carriers not affected by any cancer together with p.E134* (72.3%) represents a modest increase from 3 to 6% in BC in Group 1. 

The *XAF1* p.E134* percentage in 603 women (52.18 ± 13.38, mean age ± SD) who developed BC and were unselected for cancer history in their families are presented in Table 5 (Group 3). The genotypic frequencies for the germline variants *TP53* p.R337H and *XAF1* p.E134* were 1.32% and 1.16%, respectively, and the allele frequencies were 0.66% and 0.58%, respectively. Despite the small contribution of *TP53* p.R337H and *XAF1* p.E134* in BC development seen in Group 3, they represented increases of 4.3- and 1.5-fold, respectively. The projected percentages for all deceased women (carriers and/or noncarriers) are presented in Table 4. *TP53* p.R337H and *XAF1* p.E134* were only four and two times more frequent, respectively, than the frequencies in the normal population estimated from the Brazilian ABraOM and SELA databases [31] and from the second neonatal screening [8]. Conversely, more than 98% of all BC cases were not associated with those genetic variants.

### 3.6. Body Mass Index (BMI) in Breast Cancer Women

**Figure 3:**
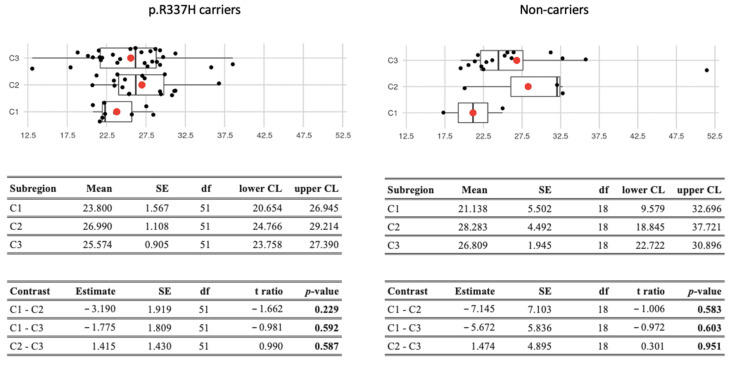
Mean (red dots) and median (vertical lines) BMIs are shown in boxplots. Estimated marginal means from a linear model (analysis of variance) detected lower and upper confidence limits (CL), and the p values between two subregions (contrast). SE, standard error; df, degree of freedom.

We did not find a significant difference in BMI between p.R337H carrier and noncarrier women from the neonatal screening cohort (Figure 3), nor between the subregions C1, C2, or C3 where they live. Women who developed BC prior to the 2006 neonatal screening (or before family enrollment to this cohort) were not included in this analysis.

## 4. Discussion

Evidence of BC associated with environmental hazards among carriers of tumor-suppressor variants has not been reported. In this study, we evaluated the impact of *TP53* and *XAF1* variants and three different environmental profiles underlying BC predisposition in women. The identification of an extended chromosome 17p13 haplotype harboring the p.E134* variant co-segregating with the p.R337H allele supported the concept of a cooperating genetic modifier in a positive feedforward loop with p53 [8]. However, *XAF1* p.E134* does not seem to be an important modifier of *TP53* p.R337H-driven breast carcinogenesis because only 77% (Group 1, equally distributed in the three subregions, 76% in C1, 79% in C2, and 75% in C3) and 75% (Group 3) of the BC cases harbored both variants, whereas newborns with unknown cancer history presented 69% with both haplotypes [8] and 72.3% of R337H carriers together with p.E134* in non-affected participants (i.e., any cancer) in the present study. The *TP53* p.R337H founder allele identified in 204 cases of different types of cancer harbored the *XAF1* p.E134* variant in 161 cases (79%), but it was identified in more than 90% of sarcomas [8]. 

BC is the most prevalent cancer in the Paraná state neonatal p.R337H cohort [3] and São Paulo state hospital-based p.R337H cohort [32]. A large percentage of our cohort families (~40%) had either only two, one, or no cases of cancer [3], or did not fit the modified criteria for Li–Fraumeni syndrome [14]. By contrast, in individuals with *TP53* gene mutations with a higher penetrance for all types of cancer in classic LFS carriers, the lifetime cancer risk was estimated at over 70% in men and nearly 100% in women, possibly due to BC cases [33,34,35,36,37]. In addition to p.R337H hypomorphic activity with low BC risk in comparison to LFS inactivating mutations, the large variation in p.R337H penetrance among the families may also be associated with study design differences, particularly between hospital-based datasets [32,38] and population-based datasets from neonatal screening [3,4,5]. We hypothesize that other unknown causes (mainly environmental hazards) are contributing to large differences in BC risk associated with p.R337H.

Early-onset breast cancer is very common in *TP53* variants presenting in classical LFS families [39,40]. Olivier et al. [40] have reported 151 BC cases in women (83 families), with more than 80% of them diagnosed at <45 years (median age 34.5 years), whereas the hypomorphic p.R337H in the present study showed a lower prevalence in all subregions (53% in C1, 44% in C2, and 49% in C3). In 214 French families harboring 133 distinct *TP53* alterations, including higher-penetrance mutations in LFS, the mean age of BC onset was younger than 45 years [39]. Conversely, an increased incidence of BC at a younger age was well-documented in a cohort of 95,256 female nurses in the USA who were followed for 16 years, and the findings were hypothesized to be associated with increased weight gain [41]. The age of BC onset in p.R337H noncarriers identified 58–64% at ≥45 years in our cohort of neonatal screening and 78% in Group 3 (non-p.R337H patients from the hospital database, not evaluated in relation to subregions C1–C3). The mean age of BC onset in wild-type *TP53* patients in other countries was 63.1 [40], which is consistent with the lower percentage (22%) found in the <45 years age group in our Group 3 noncarriers. Our hypothesis is that environmental pollution, alongside *TP53* variants, may contribute to BC onset (prevalence of 44–53% at <45 years in p.R337H carriers); however, further studies are needed to confirm this hypothesis, also taking into consideration the prognosis of molecular subtypes of BC (study in preparation).

Carriers and noncarriers were diagnosed at a younger age in the most recent or last generations, suggesting possible associations with BC screening, improved health services, and a potential increase in exposure to toxic industrial chemicals. Analysis of a cohort with 14,672,409 cases of cancer found that six types of cancer showed increased rates related to obesity among young adults (25–49 years), but this phenomenon was not observed in patients with BC [42]. However, other studies have confirmed that a high BMI contributes to BC development in postmenopausal women [43]. Women with BC admitted to a hospital in C1, coming mainly from C1 and C2 (age 40–69 years, mainly Caucasian), showed overweight (38.7%) and obesity (24.1%) [44], suggesting a modest contribution among women living in these two subregions with predominant agricultural activities. The cancer surveillance program (only for adrenocortical carcinoma) for children in the Group 1 families is also committed to promoting healthier practices in lifestyle to change diet, promote exercise, and prevent exposure to polluted environments for all adults. Overweight and obesity would more likely involve other complex mechanisms that could change the effect of the genetic variants and the environmental impact on BC risk and outcome. Further studies would be necessary to address the relationship between BMI, tumor-suppressor, and environmental factors.

In this study, we demonstrated that environmental factors may contribute more significantly to increased BC incidence and mortality rate and may cooperate with endemic *TP53* p.R337H-driven reductions in tumor-suppressor activity. BC development was less frequent in women living in regions with lower pesticide exposure and fewer industrial installations (the C2 subregion, rich in family agriculture). The strongest agricultural output previously detected in subregion C1 was associated with high DDT, DDE, DDD, and chlorine anion levels in catchment basins [19]. By contrast, the very low or undetectable levels of DDT associated with very high levels of chlorine anions found in subregion C3 are derived from industry installations plus the extensive use of chlorine-containing cleaning products in large urban areas [19]. Thus, C2 is thought to be less polluted than C1 or C3, consistent with the lower BC mortality rate found among residents of C2 in the current study. However, the number of BC cases among p.R337H noncarriers was not sufficiently large to differentiate risk among the three subregions. Inappropriate DDT use, which can contaminate drinking water and food, has been reported in several continents [45,46,47]. However, three meta-analyses showed no association between DDT exposure and increased BC risk [48,49,50]. All subregions, particularly C3, may also be affected by higher levels of other toxic chemicals released from industry installations or other types of toxic chemicals in large urban areas. 

Variations in cancer frequency illustrate the need to characterize other causes and to elucidate the complex mechanisms of cancer formation, which may involve processes initiated during embryogenesis or before conception and acquisition of subsequent gene alterations in target tissues [51]. The environmental paradigm was evaluated in this study as the main deterministic view to explain changes in the incidence of BC in Paraná (C1), which is becoming a worldwide leader in crop productivity (tons/km^2^; western and northern Paraná), in contrast to C3, which encompasses large and more industrialized cities (eastern Paraná). From 2000 to 2010, pesticide use increased by 93% worldwide and 190% in Brazil [19]. Large changes among C1, C2, and C3 were associated with significant increases in chlorine anion levels in Paraná catchment basins between 1996 and 2010, which are associated with OC use; however, other types of pesticides (the “legal” ones) were not evaluated. The authors detected OCs at high levels in the catchment basins in the C1 subregion, which is inhabited by approximately 30% of the Paraná state population and 30% of the p.R337H neonatal cohort (Group 1). In addition, the observed higher chloride levels may also reflect prior exposure to chloride-containing toxic chemicals, which are metabolized and release HCl and chlorine anions in water [19]. Our current hypothesis combining congenital malformations [19] and BC, influenced by one or more causes, such as environmental factors (e.g., DDT residues and other OCs), and acting under different induction and latency periods, is consistent with the reported association between maternal cancer and congenital malformations in a Danish nationwide cohort study [52]. Cancer risk attributed to a less-penetrant germline mutation (e.g., p.R337H) without considering possible associations with toxic environment or other mutations may result in different interpretations. This is most probably not the case for most DNA binding domain mutations in p53 (e.g., R175H, R248Q, R248W, S241F, R249S, R273C, and R280K) leading to classic LFS [53], where cancer occurs independently of environmental conditions and other genetic or epigenetic variants. Our hypothesis is, for less-penetrant germline p53 mutations, with variable cancer history (e.g., pR337H), the polluted environment and/or association with other germline and/or somatic mutations have a higher impact on BC burden. That seems to be the case for the higher BC risk observed in the p.R337H carriers of the C3 subregion than C2. 

This study has some limitations. We did not assess environmental factors in the blood of BC patients or account for known lifestyle risk factors (diet, exercise, occupation, or other types of exposures). The population growth in the three regions was stable between 2005 and 2015, yet we did not determine BC incidence compared with the mortality rate in each subregion. The death certificate is required by law, but because healthcare providers are not required to report breast cancer cases, we were unable to calculate BC incidence by subregion. Our data did not allow for multivariate analysis of environmental and genetic causes of BC; however, we can estimate that > 98% of all BC cases were not associated with the genetic variants studied here. Although the observed earlier age of BC onset in subsequent generations could suggest genetic anticipation, important bias was not avoided in the current study, limiting support for an anticipation hypothesis. Major factors opposing the occurrence of genetic anticipation include the nature of the cohort surveillance protocol, which was strongly based on two surveillance programs for earlier pediatric adrenocortical carcinoma detection [3,4,5] and family referral to other cancer specialists, improvements in health services and breast screening, and early BC onset among noncarriers.

## 5. Conclusions

*TP53* p.R337H was found to increase BC risk (4.5-fold higher than p.R337H noncarriers) and was influenced by the environment, such as in C1 and C3, and may be mildly influenced by co-segregation with *XAF1* p.E134*. We hypothesized that BC risk may be significantly increased by chemicals released through industry-related activities, chlorine-containing pesticides, or other pesticides, providing translational research opportunities to improve surveillance, particularly in regions in which the population of germline p.R337H variant carriers is high.

## Figures and Tables

**Table 1:** Distribution of p.R337H noncarrier (*n* = 3562) and p.R337H carrier women (*n* = 1096) and BC (30+ years old) in the three subregions (C1, C2, and C3) of Paraná state and according to their position in the pedigree.

		p.R337H Carrier and Noncarrier Women							
Pedigree		C1		C2		C3		Total	
Generation		Non ^a^	p.R337H ^b^	Non ^a^	p.R337H ^b^	Non ^a^	p.R337H ^b^	Non ^a^	p.R337H ^b^
I		82	14	200	37	135	27	417	78
II		292	82	601	180	450	158	1343	420
III	III ^c^	307	102	691	218	524	190	1522	510
IV		54	11	139	39	80	34	273	84
V		1	0	6	3	0	1	7	4
		**N (%)**							
BC		14 (1.9)	17 (8.1)	23 (1.4)	25 (5.2)	24 (2.0)	39 (9.5)	61 (1.7)	81 (7.4)
Total		736	209	1637	477	1189	410	3562	1096

^a^ Non, noncarrier of p.R337H. ^b^ p.R337H, *TP53* p.R337H carrier women. ^c^ The last three generations were grouped as generation III.

**Table 2:** Prevalence and risk ratio of developing BC in three subregions (C1, C2, and C3) of Paraná state among p.R337H carrier and noncarrier women.

Breast Cancer							
	*n* ^a^	Prevalence (/1000)		*n*	Prevalence (/1000)	Risk Ratio	*p* Value
							*X* ^2^
**Non-p.R337H**							
C1	14	19.02	C2	23	14.05	1.354 (0.701–2.616)	0.468
C3	24	20.19	C2	23	14.05	1.437 (0.395–1.227)	0.267
C3	24	20.19	C1	14	19.02	1.061 (0.552–2.038)	0.992
**p.R337H**							
C1	17	81.34	C2	25	52.41	1.552 (0.857–2.812)	0.200
C3	39	95.12	C2	25	52.41	1.815 (1.118–2.946)	0.020
C3	39	95.12	C1	17	81.34	1.169 (0.678–2.016)	0.677
**Total**							
C1	31	32.8	C2	48	22.71	1.445 (0.926–2.255)	0.133
C3	63	39.4	C2	48	22.71	1.735 (1.199–2.512)	0.004
C3	63	39.4	C1	31	32.80	1.201 (0.787–1.832)	0.457

^a^ *n*, Number of breast cancer cases.

**Table 3:** Age at diagnosis of breast cancer cases among p.R337H carriers and noncarriers per subregion.

**Group 1 BC Patients (Cohort from Neonatal Screening)**				
Subregion	Carriers < 45 years	Carriers ≥ 45 years	Noncarriers < 45 years	Noncarriers ≥ 45 years
	*n* = 81 BC		*n* = 61 BC	
C1: BC (%)	9 (53) ^1^	8 (47) ^1^	6 (42) ^4^	8 (58) ^4^
C2: BC (%)	11 (44) ^2^	14 (56) ^2^	8 (34) ^5^	15 (64) ^5^
C3: BC (%)	19 (49) ^3^	20 (51) ^3^	10 (41) ^6^	14 (59) ^6^
**Group 3 BC Patients (Hospital Database)**				
	Carriers < 45 years	Carriers ≥ 45 years	Noncarriers < 45 years	Noncarriers ≥ 45 years
	*n* = 8		*n* = 506 *	
Total (%)	3 (38) ^7^	5 (62) ^7^	114 (22) ^8^	392 (78) ^8^

* available from a total of 603 unselected patients. ^1,2,3,4,5,6,7,8^ The individual differences in each of these eight pairs lack power to be statistically evaluated.

**Table 4:** Mortality rate and municipalities in the subregions of Paraná state (2005–2015).

Mortality Rate								
	BC (*n*) ^a^	AASMR ^b^ (/100,000)			BC (*n*)	AASMR (/100,000)	Relative Risk	*p* Value
								*X^2^*
C1	2220	26.59		C2	2761	24.28	1.095 (1.036–1.158)	0.002
C3	2833	30.98		C2			1.276 (1.211–1.345)	<0.001
C3				C1	2220	26.59	1.165 (1.102–1.232)	<0.001
Municipalities in each subregion								
Subregions			C0 ^c^		C1	C2	C3	Total
Municipalities (*n*)			66		114	200	19	399
Women over 30 years of age in 2010			152,606		759,083	1,033,835	831,323	2,776,847

^a^ BC cases in the period (2005–2015). ^b^ AASMR, average age-specific mortality rate. ^c^ Municipalities in subregion C0 (66/399, 16.5%) were not included in the current study.

**Table 5:** *TP53* p.R337H and *XAF1* p.E134* variant allele frequencies in Group 3 (*n* = 603).

Detected Alleles	p.R337H+/ p.E134*-	p.E134*+/ p.R337H-	p.R337H+/ p.E134*+	All p.R337H+	All p.E134*+
***n* (%)**	2 (0.166)	1 (0.083)	6 (0.497)	8 (0.663)	7 (0.580)

## Data Availability

Group 3 data from women admitted with breast cancer to the Erasto Gaertner Hospital (EGH) in Curitiba (2015–2018) may be obtained upon submitting a request to EGH. Mortality data (Group 2) will be available by assessing the DATASUS registry (http://tabnet.datasus.gov.br/cgi/tabcgi.exe?sim/cnv/obt10pr.def). Group 3 data, women from 373 families identified with the *TP53* p.R337H variant, may be obtained upon submitting a request to Pelé Pequeno Príncipe Research Institute.
